# The macrophage: a therapeutic target in HIV-1 infection

**DOI:** 10.1186/2052-8426-2-10

**Published:** 2014-04-02

**Authors:** Amit Kumar, Georges Herbein

**Affiliations:** Department of Virology, UPRES EA4266 Pathogens & Inflammation, University of Franche-Comte, SFR FED 4234, F-25030 Besançon, France; Department of Virology, Hôpital Saint-Jacques, CHRU Besançon, 2 place Saint-Jacques, F-25030 Besançon cedex, France

**Keywords:** HIV-1, Macrophages, Nef, Tat, Vpr, Antiretroviral therapy, Latency

## Abstract

Human immunodeficiency virus (HIV) is still a serious global health concern responsible for more than 25 million deaths in last three decades. More than 34 million people are living with HIV infection. Macrophages and CD4+ T cells are the principal targets of HIV-1. The pathogenesis of HIV-1 takes different routes in macrophages and CD4+ T cells. Macrophages are resistant to the cytopathic effect of HIV-1 and produce virus for longer periods of time. In addition, macrophages being present in every organ system thus can disseminate virus to the different anatomical sites leading to the formation of viral sanctuaries. Complete cure of HIV-1 needs better understanding of viral pathogenesis in these reservoirs and implementation of knowledge into robust therapeutic products. In this review we will focus on the unique relationship between HIV-1 and macrophages. Furthermore, we will describe how successful antiretroviral therapy (ART) is in suppressing HIV and novel molecular and cellular strategies against HIV-1 in macrophages.

## Introduction

Human immunodeficiency virus type 1 (HIV-1) can infect several types of immune cells, however macrophages and CD4+ T lymphocytes cells are the principal targets of HIV-1 in human body [1, 2]. Macrophages are terminally differentiated immune cells which play an important role in the clearing of pathogens and cellular debris by phagocytosis. Besides, they also act as the antigen presenting cells and present processed pathogen antigen peptides to the CD4+ T cells via MHC II pathway [3, 4]. This exchange of information between macrophages and CD4+ T cells also has important role in the transmission of HIV-1 from macrophage to CD4+ T cells [5–7]. In addition, HIV-infected macrophages release soluble cytotoxic factors that can promote the apoptosis of bystander cells for example CD4+ and CD8+ T cells [8, 9].

HIV-1 infection results in the lysis of T lymphocytes (CD4+ T and CD8+ T cells) leading to their depletion, a hallmark of HIV-1 pathogenesis. On the contrary, macrophages are relatively less prone to the cytopathic effect of the virus [10, 11]. Since the life span of HIV-1 infected macrophage is long, thus they act as a source of virus production for longer period of time in infected patients [12]. In addition, macrophages are virtually present in every organ system (although with different names), thus can disseminate HIV-1 throughout the body of infected persons including brain [13]. Therefore, how HIV-1 interacts with macrophages and governs its life cycle in macrophage environment is very important. In this review we will summarize the interplay of HIV-1 and macrophages and therapeutic interventions against HIV-1 in macrophages.

## Review

### HIV-1 replication in the macrophage

#### HIV-1 entry into macrophages

**Figure 1:**
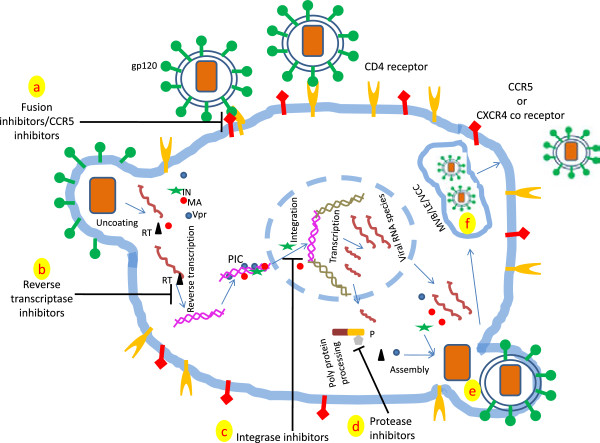
**Depicting key events of HIV-1 life cycle targeted by anti-retroviral drugs.** The anti-retroviral drugs target four critical steps of the viral life cycle which are fusion (or entry) of virion in the susceptible cell, reverse transcription, integration of proviral DNA into host chromatin and polyprotein processing by viral encoded protease. Depending upon the steps they target, the anti-retroviral drugs are termed as fusion (entry) inhibitors **(a)**, reverse transcriptase inhibitors **(b)**, integrase inhibitors **(c)** and protease inhibitors **(d)**. Targeting single step at a time usually results in the emergence of resistant mutants. ART is formulation of these inhibitors which suppresses HIV-1 growth to a significant extent. Please note that virus assembly in macrophages takes place at both plasma membrane **(e)** as well as in virus containing compartments **(f)**[47]. Only key proteins involved in HIV-1 life cycle in macrophage have been shown. Abbreviations: RT- reverse transcriptase, MA- matrix protein, IN- Integrase, Vpr-Viral protein R, P- virus encoded protease, PIC- Pre-integration complex, MVB- multi vesicular bodies, LE- late endosomes and VCC- virus containing compartment.

First step of HIV-1 entry into target host cells involves virus ligand (virus surface glycoprotein gp120) and its interaction with CD4 receptor which is present in both T cells as well as in macrophages [14, 15] (Figure 1). Second step involves the fusion of viral envelope with host cell membrane which is governed by the engagement of the co-receptors (CCR5 or CXCR4) (Figure 1). Earlier it was believed that macrophages have CCR5 receptor and most of the T cells have CXCR4 receptor resulted in macrophage tropic and T cell tropic HIV-1 terminology [1]. Further studies revealed that both the co-receptors are present on macrophages as well as in T cells *in vivo*[1, 11, 16, 17]. Notably the naturally transmitted HIV-1 viruses utilize CCR5 for their infection, even though their primary targets are T cells not macrophages. In CNS, microglia (resident macrophages of the brain) are infected via CCR5 co-receptor. Common consensus is that these R5 and X4 viruses can replicate in both macrophages as well as in T cells. However, their replication efficiency varies in cell types which depend upon the cellular environment. Furthermore, viral progeny from macrophages and T cells can be identical however, they may have different sets of host protein incorporated in their viral particle (reviewed comprehensively in [1]).

### Reverse transcription and host restriction factors

Whether HIV-1 enters via CXCR4 or CCR5 coreceptor, in both cases the viral ribonucleoprotein complex is released into the cytoplasm [11, 18] where virus encoded reverse transcriptase using viral genomic RNA as template, generates single stranded cDNA followed by double stranded (ds)DNA [19, 20] (Figure 1). However, the rate of reverse transcription is slower in macrophages than what is observed in T cells. Macrophages being terminally differentiated non dividing cells have limited dNTP pools for proviral DNA production [21, 22]. Several reports have shown that addition of deoxynucleosides to the primary human macrophage culture remarkably enhances the rate of HIV-1 reverse transcription proving that dNTP pool is an important rate limiting factor in macrophages [21, 23, 24].

Additionally, macrophages possess certain inhibitory factors which interfere with viral life cycle and are termed as host restriction factors [25, 26]. These host restriction factors include tetherin, APOBEC3G and recently identified sterile alpha motif (SAM) domain and HD domain-containing protein 1 (SAMHD1) [25–27]. APOBEC3G is known to trigger G-to-A hypermutation in nascent DNA. Tetherin (also called CD317/BST-2) hinders the release of viral progeny from infected cells [26]. HIV-1 employs several strategies to overcome these restriction factors (reviewed in [28, 29]). HIV-1 accessory protein Vif and Vpu counteracts the APOBEC3G and tetherin respectively [4, 25, 26]. Even there are reports describing tetherin antagonism by HIV-1 Nef protein [30, 31].

SAMHD1 is a macrophage specific host restriction factor which has triphosphohydrolase activity resulting in hydrolysis of dNTPs into nucleosides and triphosphates. Thus SAMHD1 reduces the dNTPs pool in macrophages to a certain level resulting in the inefficient reverse transcription of HIV-1 genomic RNA into proviral DNA [32]. However, Vpx protein of HIV-2 induces proteasome-dependent degradation of SAMHD1 through CRL4DCAF1 E3 ubiquitin ligase [27]. Recently McKnight research group, in order to search for host restriction factors, screened several human genes and identified 114 genes with significant impact on HIV-1 replication. Furthermore, their studies revealed that inhibition of all members of PAF1 family resulted in increase in HIV-1 replication. Notably PAF1 is not restricted to macrophages only, they are also expressed in primary monocytes and T-lymphocytes, suggesting exhaustive list of restriction factors against HIV-1 [33]. Recently Allouch and colleagues showed that cyclin-dependent kinase inhibitor p21 inhibits HIV-1 replication in monocyte-derived macrophages (MDMs) by interfering with reverse transcription of the viral genome by a mechanism independent of SAMHD1. Additionally, they demonstrated that p21 curtails the dNTP synthesis through the down regulation of the expression of RNR2 (a subunit of ribonucleotide reductase) necessary for the biosynthesis of dNTPs [34].

### Nuclear transport

Newly synthesized HIV dsDNA is imported to the nucleus as pre-integration complex (PIC) (Figure 1). Unlike T cells, in macrophages PIC transport to the nucleus is independent of cell division. PIC comprises of viral proteins which includes reverse transcriptase, Vpr, integrase (IN), matrix (MA, p17) and capsid protein (CA) in addition to newly synthesized dsDNA. However, CA dissociates from PIC prior to the nuclear entry. Vpr, IN and MA direct the transport PIC through nuclear pore mediated by importin α/β [35, 36] (Figure 1). However, precise function of these proteins in PIC nuclear transport is still a matter of debate [11]. Unlike IN and MA, Vpr lacks nuclear localization signal [37, 38]. In addition, interaction between importin α and Vpr is critical not only for the nuclear transport of PIC but also for the replication of HIV-1 in macrophages [39]. Furthermore, in primary macrophages, host cell protein emerin (an integral nuclear inner membrane protein) plays an indispensible role in integration of viral DNA into the chromatin [40, 41]. Primary macrophages lacking emerin have poor rate of HIV proviral DNA integration into the host chromatin however, lack of emerin does not inhibit PIC entry into the nucleus [40]. In addition, binding partners of emerin, the LEM (LAP2 (lamina-associated polypeptide 2)/emerin/MAN1) is necessary for the interaction of viral cDNA with emerin and capability of emerin to support HIV-1 infection in macrophages [40]. However, Shun and colleagues demonstrated that HIV-1 can efficiently infect dividing cells despite of the absence of emerin, suggesting the role of emerin in HIV-1 infection restricted to only macrophages [42]. Besides several other host factors are involved in the HIV life cycle in macrophages have been reviewed recently [43].

### HIV-1 transcription

HIV-1 transcription is governed by binding of viral proteins and host factors to the long terminal repeat (LTR) of the virus, which functions as viral promoter [44]. Host factors include nuclear factor kappa B (NF-κB) family, AP-1 (activator protein 1), Sp family, C/EBP (CCAAT enhancer binding protein and NFAT (nuclear factor of activated T cells). These host factors have specific binding sites present on LTR. On the other hand, viral proteins Tat and Vpr also bind to the LTR to govern HIV-1 transcription [20, 44]. Worth mentioning, host factors could be cell type specific, for example C/EBP proteins and their binding sites are critical for HIV-1 replication in macrophages but not in CD4+ T cells [45]. In addition, primary macrophages infected with HIV-1 having mutation in C/EBP binding sites does not support HIV-1 replication. On the other hand, primary CD4+ T cells, Jurkat and H9 cells support the replication of HIV-1C/EBP mutants [45].

### HIV-1 assembly in macrophages

In case of primary CD4+ T cells, HIV-1 assembly takes place at the plasma membrane [46]. On the other hand, the corresponding site in macrophages is not yet fully characterized [47]. Initial studies demonstrated the presence of HIV-1 virion particles in multivesicular bodies (MVBs) or late endosomes (LEs) like structures [47, 48] (Figure 1). Even immuno-electron microscopy studies supported latter finding as their studies revealed the presence of MVB specific markers (for example CD53, CD9, tetraspanins, CD81 and MHC II) in those structures [47, 49–51]. In addition, HIV-1 progeny released from infected macrophages also possess these markers, further strengthening the view that macrophages are released from LEs or MVBs [47, 50, 52]. However, several studies revealed that structures harboring HIV-1 in infected macrophages have some distinct characters which are not characteristics of LEs or MVBs. These unique characteristics include tubular connection to the extracellular space and neutral pH [53]. The term ‘virus containing compartments’ (VCCs) has been assigned to the structures which act as the site for the virus assembly in macrophages [47] (Figure 1). Interestingly, these VCCs are also present in uninfected macrophages however, they become more prominent upon HIV-1 infection [51, 53]. Worth mentioning, VCCs have limited access to the innate and adaptive immune effector molecules [47]. In contrast, several studies are in the favor of budding of HIV-1 progeny from plasma membrane in infected macrophages [54]. Taken together, these contrasting studies indicate that there is a fair possibility that HIV-1 may bud from plasma membrane as well as from VCCs (Figure 1). VCCs may act as a safe house for HIV-1 in macrophages leading to HIV-1 reservoirs. However, elegant experiments are further required to support this hypothesis.

## Interplay between HIV proteins and cell signaling in macrophages

Among HIV-1 proteins, the viral proteins Tat, Vpr and Nef interfere with signaling pathways in macrophages.

### Tat

The trans-activator of transcription (Tat) protein is a 86–101aa virus encoded pleiotropic protein which directly or indirectly modulates several steps of HIV life cycle including replication, transcription and progeny release by regulating both cellular as well as viral gene expression [20, 55–57]. In addition, Tat has been detected in sera of HIV infected patients as well in cell culture settings indicating its role as a modulator of cellular function in infected cells and also to target bystander cells [20, 58]. Furthermore, monocytes, macrophages and microglia are activated by Tat protein [20]. In addition, Tat is known to trigger the expression of HIV coreceptors (CXCR4, CCR5 and CCR3) in macrophages in a dose-dependent manner which might positively influence HIV-1 infection [59]. Furthermore, Tat acts as a potent chemoattractant for monocytes, macrophages and dendritic cells [60, 61]. Tat induces the production and release of tumor necrosis factor alpha (TNF-α) from macrophages [62]. Further, Tat mediated TNF-α induction was NF-kappa B (NF-κB) dependent and mediated through activation of signaling cascades including PLC (phospholipase C), protein kinase A and protein tyrosine kinase [20]. In addition, Tat enhances the endogenous levels of Ca^2+^ in macrophages which may subsequently lead to the production of chemokines and pro-inflammatory cytokines [63]. Latter events may be responsible for HIV-1 induced neuropathogenesis and inflammation [64].

### Viral protein R (Vpr)

Vpr is a virion-associated protein dispensable for viral replication in T cells however is indispensible for viral replication in macrophages [65]. Vpr has been localized in cytoplasm as well as in nucleus of the infected cells [66]. Vpr is a multifunctional protein which regulates viral replication, cellular events like NF-κB-mediated transcription, apoptosis and cytokine production [20, 67]. Effect of recombinant Vpr (rVpr) has been demonstrated in macrophages. Although high concentration of rVpr resulted in significant cytotoxicity in macrophages however, at lower concentration rVpr has been shown to increase the biological activity of several transcription factors including NF-κB, c-Jun and AP-1 in promonocytic cells and primary macrophages [68]. In addition rVpr stimulates HIV-1 replication in acutely infected primary macrophages. Furthermore, infection of macrophages with Vpr-deficient viral mutants resulted in decreased production of p24 which can be corrected by addition of rVpr [69]. Moreover, Vpr independently enhances the expression of cyclin-dependent kinase inhibitor 1A (CDKN1A/p21) in macrophages whereas Vpr mutants exhibit lack of upregulation of p21 and display reduced viral replication [70]. Taken together, data strongly suggest that Vpr enhances the viral replication in acutely and latently infected macrophages.

### Nef

Nef is expressed during early life cycle of HIV-1. Nef is a 27 kDa myristoylated protein required for efficient viral replication in infected cells [71, 72]. In addition, Nef enhances the survival of infected cells which helps in the expansion of infectious viral population. Furthermore, Nef hampers the immune system of infected patients by several mechanisms including down-regulating the expression of MHC I, MHC II, CD28, CD4 [73, 74] and by activating PI3K [75]. Nef down-regulates the expression of CD4 receptor in macrophages which serves two purposes. Firstly, CD4 down-regulation in infected cells may promote the release of viral progeny by avoiding sequestration of viral envelope by CD4 [76]. Secondly, it helps in avoiding superinfection which otherwise could lead to premature cell death [71, 76].

In monocyte derived macrophages (MDMs) exogenously added recombinant Nef (rNef) regulates the expression of several genes in a short time span (2 hours). These findings indicate a robust transcriptional programming governed by Nef protein leading to the production and secretion of soluble factors which in turn activates STAT1 and STAT3 in primary monocytes/macrophages [20, 77]. Similarly, addition of rNef to the MDMs cultures resulted in the rapid induction of transcription factors NF-κB, AP-1, and c-Jun N-terminal kinase and enhanced HIV-1 transcription. Furthermore, *in vitro* treatment of macrophages with rNef has been reported to trigger IKK/NF-κB, MAPK and IRF-3 signaling cascades. Additionally, Nef induces robust phosphorylation of MAPKs, including ERK1/2, JNK, and p38 [20, 78]. Notably, the role of Nef in HIV-HCV coinfected macrophages has been recently described [79].

## Contribution of macrophages to HIV-1 pathogenesis

**Figure 2:**
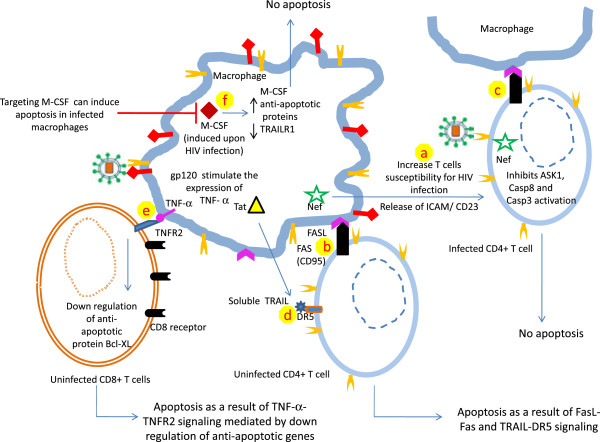
**Relationship between macrophages and T lymphocytes in HIV-1 infection.** Macrophages harboring HIV-1 play an important role in HIV pathogenesis. Nef stimulates the release of soluble factors ICAM and CD23 which makes uninfected CD4+ T cells more susceptible to HIV infection, thereby favoring the expansion of the viral reservoir **(a)**. In addition, Nef induces the expression of Fas ligand (FasL, CD95L) on HIV-infected cells. Interaction of CD95L and its receptor (Fas) present on uninfected CD4+ T cells results in apoptosis **(b)**. On the other hand in infected CD4+ T cells, Nef inhibits the expression of proteins involved in apoptosis including ASK1, caspase 8 and caspase 3 **(c)**, protects infected CD4+ T cells from cell death and further expands the viral reservoir. HIV regulatory protein Tat stimulates the production and release of TRAIL from the infected macrophages. TRAIL binds with its receptor (DR5) present on uninfected CD4+ T cells and induces apoptosis **(d)**. Furthermore, gp120 interaction with CXCR4 receptor increases the expression of TNF-α on macrophages which interacts with TNFR2 present on CD8+ T cells. This interaction results in the down regulation of the anti-apoptotic protein Bcl-XL and ultimately leads to apoptosis **(e)**. Moreover, HIV infection in macrophages is known to induce macrophage colony stimulating factor (M-CSF) which inhibits the expression of TRAILR1 on macrophages and upregulates the expression of anti-apoptotic proteins **(f)**, favoring the resistance to apoptosis of infected macrophages. Therefore, targeting M-CSF has been suggested to increase apoptosis in infected macrophages.

HIV-1 pathogenesis is characterized by progressive cell depletion involved in adaptive immunity including CD4+ T and CD8+ T cells [8, 9]. Not only HIV-infected CD4+ T cells are lysed but uninfected CD4+ T cells more prominently undergo apoptosis [80] (Figure 2). Nef plays dual role in HIV-1 pathogenesis. On one hand, Nef protects HIV-infected cells from cell death to favor efficient viral production. On the other hand, Nef induces apoptosis in bystander CD4+ T cells. Furthermore, it has been shown that Nef-expressing macrophages release paracrine factors including soluble ICAM and CD23 which increase the lymphocytes permissively for HIV-1 infection [81] (Figure 2). Additionally, Nef induces the expression of Fas ligand (CD95L) on the surface of infected T cells. Furthermore, interaction between CD95L and its receptor present on cells in close vicinity triggers apoptosis in bystander cells [8, 82] (Figure 2). Notably, Nef protects infected cells from apoptosis via CD95-CD95L *cis* interaction by inhibiting ASK1 (apoptosis signal-regulating kinase 1), caspase 8 and caspase 3 activation [20, 83] (Figure 2). Worth mentioning, ASK1 is a common partner of Fas and TNF-α mediated death signaling cascades [83].

In addition, uninfected macrophages have been shown to confer resistance against apoptosis in productively infected CD4+ T cells. Although expression of Nef by these infected CD4+ T cells is necessary for anti-apoptotic behavior however, presence of macrophages further enhances the number of non-apoptotic cells via intercellular contacts mediated by TNF stimulation [84]. This may be the one of the mechanisms of promotion of HIV-1 reservoir in T cells by macrophages. Another regulatory protein of HIV, Tat has been reported to stimulate the expression of TRAIL TNF related apoptosis-induced ligand (TRAIL) in U937, monocytes and primary macrophages [85, 86], which results in the apoptosis of uninfected cells (Figure 2). This finding provides an insight into another mechanism of elimination of bystander cells.

Recombinant glycoprotein gp120 (rgp120) (from X4 strain) has been reported to induce apoptosis of cytotoxic T cells (CTLs, CD8+ T cells). Furthermore, apoptosis is mediated by interaction between TNFR-2 present on the CD8+ T cells and TNF-α bound on the surface of macrophages [9] (Figure 2). In addition, the expression of TNFR-2 and TNF-α is positively regulated by treatment with rgp120 or upon HIV infection [9]. Moreover, stimulation of TNFR-2 receptor in primary T cells resulted in the down-regulation of anti-apoptotic protein Bcl-XL which may further explain CD8+ T cell elimination [87].

These results collectively revealed that macrophages play a central role in the propagation of HIV-1 infection, in depletion of CD4+ and CD8+ T cells, and in conferring anti-apoptotic characteristics to the HIV infected cells thereby favoring the expansion of the viral reservoir.

### Macrophages and cytotoxic T cells (CTLs)

HIV-1 specific cytotoxic T cells (CTLs) play an important role in controlling HIV-1 infection during early stage of infection [88, 89]. CTLs act on the information provided by CD4+ T cells or antigen presenting cells [90]. However, in HIV-1 infected patients even effective CTLs response is also hampered. Studies showed that Nef downregulates the expression of HLA class I molecule in infected CD4+ T cells resulting in their escape from HIV-1 specific CTLs [91]. Interestingly, Fujiwara and Takiguchi, in their *in vitro* study demonstrated that HIV-1 specific CTLs are capable of effectively suppressing R5 virus replication in infected macrophages [92]. Furthermore, their data revealed that HIV-1 infected macrophages induce more proliferation of HIV-1 CTLs as compared to infected CD4+ T cells. Taken together data suggest the involvement of effective response of macrophages during early phase of HIV-1 infection [92]. However, *in vivo* the role of HIV-1 infected macrophages is largely influenced by their activation states [15]. Notably, macrophages are proposed to be in three kinds of activation states which are designated as M1 (pro-inflammatory in nature), M2 (anti-inflammatory in nature) and deactivated macrophages. Of note, M1 macrophages produce cytokines IL-23, IL-12, IL1-β, TNF-α and support Th1 response [15, 93, 94]. On the other hand, in M2 activation state, macrophages secrete IL-10 and support Th2 responses [15, 94]. According to proposed model, during early stage of HIV-1 infection, M1 activation is predominant which favors robust HIV-1 transcription and formation of viral reservoirs [15]. As the infection progressed, M1 state is off and M2 activation state is predominant followed by deactivation of macrophages resulting finally in failure in presenting antigen to the CTLs [15].

## Search for apoptosis inducing agents in HIV-infected macrophages

Induction of apoptosis in chronically infected T cells has been suggested as a possible cure for HIV infection [95, 96]. Several new targets have been suggested in T cells, alteration of which can induce programmed cell death in infected T cells [97–99]. Vigorous efforts are also required to search for similar targets in infected macrophages.

HIV-1 infection in macrophages has been reported to induce the production of macrophage colony stimulating factor (M-CSF). Furthermore, M-CSF positively regulates the expression of anti-apoptotic proteins (Bfl-1 and Mcl-1) and inhibits the expression of death receptor TRAIL-R1 (Figure 2). Additionally, targeting of M-CSF has been also reported to enhance the apoptosis in macrophages [100]. In another recent report, apoptotic effect of viral protein Vpr has been examined in MDMs and THP1 macrophages. Their finding revealed that Vpr is not able to induce apoptosis in MDMs and THP1. Unlike undifferentiated cells, Vpr does not down regulate the expression of Bcl2 and inhibitors of apoptosis (IAPs) family members in macrophages [101]. Furthermore, down regulation of IAP1 and IAP2 make the macrophages susceptible for Vpr meditated apoptosis. Altering IAP activity has been suggested as a possible way to induce apoptosis in infected macrophages [101].

## Conventional therapies against HIV-1 in macrophages

Currently, combinatorial antiretroviral therapy (ART) is widely used in suppressing HIV-1 infection to a significant level [102, 103]. ART has made a remarkable contribution in improving and enhancing life span of infected patients [104]. HIV-1 growth kinetics is different in macrophages and T cells suggesting varied impact of antiretroviral drugs against HIV-1 in these target cells. Here we will briefly describe the potential contribution of ART in HIV-infected macrophages.

### Reverse transcriptase inhibitors (RTIs)

More than 25 compounds have been licensed for treating HIV in infected patients [105]. Out of them nearly fifty percent are reverse transcriptase inhibitors (RTIs) [105]. RTIs are of two types which are nucleoside reverse transcriptase inhibitors (NRTIs) and non nucleoside reverse transcriptase inhibitors (NNRTIs) [13].

#### Nucleoside reverse transcriptase inhibitors (NRTIs)

NRTIs target reverse transcriptase enzyme which is responsible for conversion of HIV genomic RNA into cDNA, an important step in the life cycle of HIV (Figure 1). NRTIs include emtricitabine, tenofovir, abacavir, lamivudine, stavudine, zalcitabine, didanosine and didovudine [105].

NRTIs mimic and compete with natural nucleotides pool for incorporation into growing chain of nascent HIV DNA. Notably, NRTIs require intracellular phosphorylation for conversion into functional inhibitors of HIV. Since most of NRTIs lacks 3′ OH moiety, therefore their incorporation into nascent HIV DNA leads to termination of DNA chain formation. Efficacy of these NRTIs majorly depends upon the levels of dNTPs pools [13, 24]. As discussed earlier, macrophages being terminally differentiated non dividing cells have limited pools of dNTPs as compared to actively dividing cells [13, 106]. Therefore, theoretically in this scenario, NRTIs will face less competition with natural dNTPs in macrophages. That may be the one of the reasons for better efficacy of NRTIs in macrophages as compared to CD4+ T cells [21, 107, 108]. In fact NRTIs have shown promising results in reducing the neuropathological consequences of HIV encephalitis in the CNS and onset of HIV-associated dementia (HAD) [108–110]. Notably, in CNS, macrophages represent the major HIV infected population [101]. In addition, NRTIs treatments in macrophages result in fewer emergences of resistant HIV mutants as compared to lymphocytes [111].

Strikingly, NRTIs efficacy is remarkably different in acutely and chronically infected macrophages. Exact mechanism responsible for such observation is poorly understood. Since chronically infected cells possess integrated HIV DNA into host chromatin, HIV RNA produced via integrated DNA using transcription by host RNA polymerase is therefore not susceptible to NRTIs. Besides this, there must be several other mechanisms responsible for the difference in the efficacy of NRTIs between chronically and acutely infected macrophages [13, 108]. Notably, NRTIs are associated with several undesirable effects including their interference with cell cycle and mitochondrial environment and also induce apoptosis [112, 113].

#### Non nucleoside reverse transcriptase inhibitors (NNRTIs)

Licensed NNRTIs include rilpivirine, etravirine, delavirdine, efavirenz and nevirapine. Unlike NRTIs, NNRTIs do not require phosphorylation nor compete with natural dNTPs pools for their action. NNRTIs act by binding to the hydrophobic pocket near the reverse transcriptase active site resulting in the inhibition of polymerization reaction [13, 106]. Since NNRTIs efficacy does not depend upon the cellular dNTPs pools, therefore their impact on acutely infected macrophages and CD4+ T cells is not significantly different. Furthermore, macrophage colony stimulating factor which positively regulates the dNTPs pool, have no effect on the NNRTIs efficacy against HIV [106]. Notably, NNRTIs have less adverse effects as compared to NRTIs. However, Badley research group has studied the side effects of NNRTI in Jurkat T cells and PBMCs. They observed the induction of caspase and mitochondrial dependent apoptosis by NNRTIs [114].

Like NRTIs, NNRTIs anti-HIV activities remarkably differ between acutely infected and chronically infected macrophages. To be more precise, EC_50_ of NNRTIs against acutely infected macrophages varies from 10 to 50nM. On the other hand, their effect is negligible against chronically infected macrophages [13, 108]. Reasons for these observations are incompletely understood.

### Integrase inhibitors

Chronic HIV infection is mostly characterized by integration of proviral DNA into the host chromatin (Figure 1). This process called strand transfer is governed by HIV encoded enzyme called integrase and is indispensible for the establishment of latency [115, 116] (Figure 1). Till date three integrase inhibitors (raltegravir, elvitegravirs and dolutegravir) have been approved for clinical use. Efficacy of integrase inhibitors has been studied in MDMs and lymphocytes and showed similar results [117]. Notably, even single point mutation in integrase confers resistance against the integrase inhibitor raltegravir [118]. However, other integrase inhibitors are still effective in that situation [119]. Simultaneous targeting of multiple components of HIV is necessary to avoid emergence of resistant mutants.

### Protease inhibitors (PIs)

Till date 10 protease inhibitors (PIs) have been licensed for the treatment of HIV-1 infection. Unlike reverse transcriptase inhibitors, PIs act at post integration stage of HIV-1 life cycle [106] (Figure 1). HIV protease helps in the production of infectious viral progeny. PIs bind at the active site of HIV proteases and make them non functional (Figure 1). As compared to reverse transcriptase inhibitors, PIs are effective in both acutely as well as chronically infected macrophages and CD4+ T cells. However, concentration required for effective HIV inhibition is more in case of chronically infected macrophages as compared to CD4+ T cells [120, 121]. In clinical situation, bioavailability of PIs in plasma and tissue specific macrophages is considerably different. As a result, HIV in tissue macrophages may escape from PIs [106]. Furthermore, since so far no impact of PIs on integrated HIV DNA has been reported, therefore lapse of PIs treatment will rapidly result in the production and release of infectious HIV virions [106].

### Entry/fusion inhibitors

Till date, enfuvirtide and maraviroc are the two approved entry inhibitors against HIV [105]. Enfuvirtide (also called Fuzeon, T-20) is a derived from gp41 (HIV envelope protein), which inhibit hairpin formation critical for the fusion of viral envelope with host membrane [13, 106, 121] (Figure 1). Enfuvirtide inhibits HIV-1 entry into different target cells including macrophages, PBMCs and immature dendritic cells [122]. However, comprehensive studies of these inhibitors in primary macrophages are further needed.

On the other hand, maraviroc is a small molecule which binds with CCR5 receptor reversibly and prevents the virus host interactions [13] (Figure 1). Notably, maraviroc is so far the only CCR5 antagonist licensed for the treatment of HIV-infected patients [123]. Due to serious side effects and lack of clinical efficacy, other CCR5 inhibitors including aplaviroc, vicriviro and TAK-779 are no more considered for clinical development. Resistance to maraviroc has been reported [124] and responsible mechanisms have been studied [125]. New CCR5 antagonists are in different stages of development and cocktail of these CCR5 antagonists with other ART may improve the results against HIV infection.

## Novel therapeutics against HIV-1 in macrophages

Multiple novel approaches are required to completely eradicate HIV-1 from infected patients. Here we will focus on novel molecular therapeutics tools emerged against HIV-1 in macrophages.

### Carbohydrate-binding agents (CBAs)

CBAs have been described as anti-HIV molecules which specifically target glycans of HIV-1 gp120 [126, 127]. As a result of glycosylation of gp120, macrophages and dendritic cells lose their ability to recognize and present processed antigen to the CD4+ T cells to significant level, resulting in inefficient transfer of infection to the CD4+ T cells [13]. Balzarini and colleagues revealed that even brief exposure of HIV-1 to CBA hampers the ability of immature dendritic cells (having glycan-targeting C-type DC-SIGN lectin receptor) to bind HIV-1 and prevent syncytia formation when co-inoculated with T cells [128]. Recently, Balzarini research laboratory has shown that griffithsin (GRFT), an anti-HIV CBA inhibits the interaction between DC-SIGN and HIV gp120 protein and efficiently hampers the transfer of HIV-1 to CD4+ T cells [129]. Impact of CBAs in chronic HIV-1 infection is poorly defined.

### PI3K/Akt blocking agents

The PI3K/Akt signaling cascades have been widely recognized as a favorable target for anti-cancer strategies [130]. Several groups demonstrated that PI3K/Akt inhibitors in cancer therapy are well tolerated and have minimum toxicological profile in animal models and humans [131, 132]. In past few years inhibitors of PI3K/Akt signaling have been employed as anti-HIV-1 strategy. PI3K/Akt inhibitors have been shown to effectively inhibit HIV-1 replication in acutely infected primary macrophages. PI3K/Akt inhibitors used by Chugh *et al.* were optimally effective at 200 nM which is far above from physiological relevant concentrations [133]. Despite this, their results provide a valuable insight into a signaling event specifically active in HIV-1 infected cells. Additionally, the blockade of the PI3K/Akt pathway could favor apoptosis and the clearance of infected cells. The impact of PI3K/Akt inhibitors on chronically infected macrophages needs to be further investigated.

### Small interfering RNA (siRNA)

siRNAs are robust molecules which can practically degrade any viral RNA species [134]. siRNAs or shRNAs have been found to be effective in inhibiting HIV-1 replication in several cell types including primary macrophages [135]. Information of siRNAs against HIV has been compiled in the form of database called HIVsiDB [136]. HIVsiDB has information of more than 750 anti-HIV siRNAs [136]. *In vivo* toxicity, lack of effective delivery tools, generations of viral escape mutants are main hurdles in the development of siRNA as an effective therapeutic tool against HIV.

### Immune based therapeutics

HIV-1 infection ultimately results in the depletion of CD4+ T and CD8+ T cells. Efforts have been made in the direction of boosting immunity against HIV-1 [137]. For example, in various studies the application of IL-2, IL7, IL-12 and growth hormone have been reported to result in increase in CD4+ T counts in HIV-1 infected individuals [138–141]. Interestingly, IL-2 along with ART significantly reduces HIV-1 replication in infected patients as compared to ART only treated patients. However, upon treatment cessation virus bounce back indicating the inability of IL-2 to enhance immunity for the longer period of time [96, 138]. In addition, role of IL-15 has been suggested in improving functionality of anti-HIV CTLs and natural killer (NK) cells *in vitro*[142]. Moreover, IL-15 enhances simian immunodeficiency virus (SIV) specific CD8+ T cells, NK cells and decreases the number of SIV infected cells in lymph node in infected rhesus macaque [143]. Surprisingly, viral load was found to be increased more than two fold upon IL-15 treatment [143]. Notably, IL-21 treatment in SIV infected macaques resulted in increase in granzymes B and perforins in NK cells and CD8+ T cells [96, 144]. Benefits of such transient immunity evoke by interleukins and impact of continuous use of such immune based therapeutics on the health of HIV-1 infected individuals need to be carefully addressed.

### IL-27, an anti-HIV cytokine

IL-27 is a cytokine belonging to the IL-12 cytokine family and plays important roles in innate and adaptive immunity [145]. IL-27 is produced by epithelial cells, dendritic cells and macrophages [146]. Several research groups have documented the anti-HIV properties of IL-27 in MDMs, CD4+ T cells, immature and mature dendritic cells [147]. Mechanistic details of anti-HIV cytokine IL-27 have been recently revealed. IL-27 down regulates the expression of SPTBN1 (spectrin β nonerythrocyte 1), one of the host factor required for HIV-1 infection in macrophages [148]. Furthermore, IL-27 down-regulates the expression of SPTBN1 via TAK-1-mediated MAPK signaling cascade [148]. Importantly, their results indicate that SPTBN1 is a critical host component which can be targeted to inhibit HIV-1 replication in one of the principal HIV-1 reservoirs, the macrophages.

## Macrophage targeted carriers

Effective therapeutic agent must be complimented with effective delivery tools for the successful delivery of results. Nanotechnology has made it possible to deliver the therapeutic agents to specific cell types or anatomical location which otherwise are not accessible by conventional delivery methods [149]. It is assumed that anti-HIV drugs delivered via nano-carrier can be selectively accumulate in infected cell types while uninfected cells will have much lower concentration of drugs therefore, will have less side effects [150]. Wan and colleagues have developed nano-carrier based system for drug delivery in macrophages using formyl methionine-leucine-phenylalanine (fMLF) peptide-PEG derivatives [151]. fMLF are employed because fMLF receptors are specifically present on phagocytic cells including macrophages and fMLF binds to the receptors present on macrophages with high affinity [151, 152]. Bio-distribution of fMLF-PEG nano-carrier was studied *in vivo*, revealed the greater accumulation of fMLF-PEG into macrophages of kidneys, spleen and liver as compared to only PEG [152]. Results are encouraging and suggest the feasibility of specifically targeting HIV-1 reservoir in macrophages.

## Myeloid cells of central nervous system (CNS) and HIV-1

ART has significantly reduced morbidity and mortality burden associated with HIV-1. However, despite of that significant number of the patients receiving ART develops HIV-1 associated CNS disorders [153, 154]. Notably, Zink and colleagues demonstrated that ART is able to reduce the viral load in cerebrospinal fluid of macaques infected with simian immunodeficiency virus (SIV). However, they observed the presence of SIV DNA in CNS [155]. In CNS, major reservoirs of HIV-1 are the cells of myeloid origin which include meningeal macrophages, microglia and perivascular cells. Therefore, the interplay between these cells and HIV-1 is of utmost importance. Recently role of HIV-1 Tat protein has been shown in disrupting synaptical architecture *in vitro* as well *in vivo*[156–158]. Lu and colleagues have further demonstrated the involvement of CNS resident myeloid cells in deteriorating the synaptical architecture in response to Tat [157].

In addition, recently role of cathepsin B secreted by HIV-1 infected macrophages in neural apoptosis has been also described [159]. Notably, low level of cathepsin B has been detected in the post-mortem brain tissue of HIV-1 individual with HAD but not in normal individual or HIV-1 infected individual with normal cognition. Their results suggest the involvement of cathepsin B in HAD [159]. Altogether above findings provide a valuable insight into the mechanism of HIV-1 associated CNS disorder which involves myeloid cells, their secretome and viral proteins. These novel findings will help in generating new targets for managing HAD.

## HIV-1 latency in macrophages and reactivation: the “flushing out” therapy

Although highly active retroviral therapy (ART) has significantly reduced viral levels (50 copies/ml) in infected patients however, interruption of ART results in rapid increase in viremia. HIV infection leads to the rapid depletion of CD4+ T and CD8+ T cells. Despite there is certain percent of cells where virus integrate with host chromatin. These cells do not produce virus in resting condition, however produce it upon activation [160, 161]. These cells represent a pool of latent infection and are a main obstacle in complete eradication of HIV-1 from infected patients [96, 116, 162]. Besides resting CD4+ T cells, it is suggested that monocytes, macrophages, dendritic cells and hematopoietic stem cells can be latently infected with HIV [163–165]. There are experimental evidences in the support of latency in monocytes [163, 166].

Role of macrophages in dissemination of virus and expanding viral reservoir especially in T lymphocytes has been discussed elsewhere in this review. Prolonged life span and resistance to HIV cytopathic effects make macrophages as unique viral reservoirs. However, association between HIV-1 latency and macrophages is less clear. HIV infected patients on ART treatment are reported to have only few macrophages infected in lymph nodes however undergoes reactivation in case of opportunistic infections [167]. Interestingly, FDA approved amphotericin B (an antifungal drug) has been reported to reactivate HIV-1 in THP89GFP cells (a model cell line for the HIV-1 latency in macrophages) but not in T lymphocytes [168]. However, when amphotericin B induced THP89GFP cells are co-cultured with J89GFP (latently infected T cells), they activate latent HIV in latter cells [168]. In addition, recently role of polybacterial challenge in activating latent HIV-1 in the cells of monocyte/macrophage lineage has been shown *in vitro*[169, 170]. These findings indicate that macrophages may be a site of HIV-1 latent infection. Unlike CD4+ T cells, pre-integration latency in macrophages may contribute to the viral reservoir formation to a significant extent [171]. Mechanism/s responsible for post integration latency in macrophages is poorly understood. However, presence of host transcriptional repressors, anti-HIV microRNA and lack of functional Tat could play significant role in establishing post-integration latency in infected macrophage [171]. For example host factor C/EBPb is known to repressor HIV-1 transcription in macrophages which may contribute to HIV latency. In addition, in human microglial cells, CTIP2 (a highly expressed transcriptional repressor in brain) is known to inhibit the HIV-1 replication mediated by recruitment of chromatin modifying complex involving HDAC1, HDAC2 and methylase SUV39H1 [172]. Role of CTIP2 has been suggested in post integration latency in microglia cells [165, 172].

Current efforts have been made in the direction of reactivation of HIV from latent reservoir followed by their complete removal by ART [96]. According to this hypothesis, cells in which latency is reactivated should die either due to viral cytopathic effect or due to recognition by cytotoxic T cells [96, 115]. Furthermore, the fresh infection by viral progeny (released from lysed cells) will be inhibited by ART.

Several kinds of new approaches have been employed in reactivating HIV including the use of histone deacetylase inhibitors (HDACi) such as valproic acid (VPA), trichostatin (TSA), suberoylanilide hydroxyamic acid (SAHA) and sodium butyrate, methylation inhibitors including BIX-01294, 5-aza-2′deoxycytidine (Aza-CdR) and chaetocin, NFκB activators for example TNF-α and bryostatin and protein kinase C modulators and immune modulators including IL-7 and IL-15 [96, 105, 116, 173]. These new compounds have shown significant results in reactivating latency in CD4+ T cells and are at different stages of development. For example first successful clinical trial has been reported with HDACi, valproic acid (VPA) [165, 174]. However, these findings are not confirmed in other trials [175, 176].

Regarding efficacy of these novel compounds in reactivating latency in macrophages, not many reports are available. However, several HDACi have been tested in ACH2 and U1 cell lines and found to be equally effective in both cell lines [177]. Recently, Matalon and colleague tested ITF2357 (givinostat) and VPA in ACH2 and U1 cell line. Their data revealed that ITF2357 is more potent in activating latency as compared to VPA [178]. Notably, givinostat has been found to be safe in healthy individuals in phase I trial [179]. Altogether data from *in vitro* studies suggest that agents used in reactivating latency in T cells have similar effects in cells of monocyte/macrophage lineage. However, in clinical trials viral load has been mainly determined in T lymphocytes. Importantly, isolation of monocytes followed by production of monocyte derived macrophages is rather a lengthy process as compared to isolation of T lymphocytes. In addition, brain resident macrophages represent the anatomical sanctuaries where drug penetration is poor and determination of drug efficacy in these sanctuaries is rather a difficult task [180, 181]. Furthermore, the presence of efflux pumps and array of metabolic enzymes in blood brain barrier further put the efficacy of drugs in a difficult proposition. CNS resident macrophages play an important role in HAD, a severe morbidity of HIV-1 infection. Treating HIV-1 needs holistic view where besides T lymphocytes cells of monocyte/macrophage lineage must be taken into consideration. Ignoring one or other viral reservoir will not result in any favorable outcome.

## Conclusion

Macrophages are among the early targets of HIV-1. They also act as chronic and latent viral reservoirs. Although ART has suppressed viremia in most of infected patients, complete eradication is not possible without clearance of HIV-1 from latent reservoirs. Novel therapeutics options have emerged against these reservoirs. However, delivery of therapeutic molecules *in vivo* is still a major challenge. In the future, combinatorial therapies equipped with precise delivery tools can fulfill the scientific dream of the complete eradication of HIV-1 from infected patients.
